# Exercise induced hypoalgesia during different intensities of a dynamic resistance exercise: A randomized controlled trial

**DOI:** 10.1371/journal.pone.0299481

**Published:** 2024-04-16

**Authors:** Abigail T. Wilson, John Pinette, Kaitlyn Lyons, William J. Hanney

**Affiliations:** 1 School of Kinesiology and Rehabilitation Sciences, College of Health Professions and Sciences, University of Central Florida, Orlando, Florida, United States of America; 2 Musculoskeletal Research Lab, Institute of Exercise Physiology and Rehabilitation Science, University of Central Florida, Orlando, Florida, United States of America; Indiana University-Purdue University Indianapolis, UNITED STATES

## Abstract

**Introduction:**

Exercise produces an immediate lessening of pain sensitivity (Exercise-Induced Hypoalgesia (EIH)) in healthy individuals at local and distant sites, possibly through a shared mechanism with conditioned pain modulation (CPM). Dynamic resistance exercise is a recommended type of exercise to reduce pain, yet limited research has examined the effects of intensity on EIH during this type of exercise. Therefore, the primary purpose of this study is to compare changes in PPT at a local and distant site during a leg extension exercise at a high intensity, a low intensity, or a quiet rest condition. A secondary purpose is to examine if CPM changes after each intervention. The final purpose is to examine if baseline pain sensitivity measures are correlated with response to each intervention.

**Methods:**

In a randomized controlled trial of 60 healthy participants, participants completed baseline pain sensitivity testing (heat pain threshold, temporal summation, a cold pressor test as measure of CPM) and were randomly assigned to complete a knee extension exercise at: 1) high intensity (75% of a 1 Repetition Maximum (RM), 2) low intensity (30% 1RM), or 3) Quiet Rest. PPT was measured between each set at a local (quadriceps) and distant (trapezius) site during the intervention. CPM was then repeated after the intervention. To test the first purpose of the study, a three-way ANOVA examined for time x site x intervention interaction effects. To examine for changes in CPM by group, a mixed-model ANOVA was performed. Finally, a Pearson Correlation examined the association between baseline pain sensitivity and response to each intervention.

**Results:**

Time x site x intervention interaction effects were not significant (F(5.3, 150.97) = 0.87, p = 0.51, partial eta^2^ = 0.03). CPM did not significantly change after the interventions (time x intervention F(1,38) = 0.81, p = 0.37, partial eta^2^ = 0.02. EIH effects at the quadriceps displayed a significant, positive moderate association with baseline HPT applied over the trapezius (r = 0.61, p<0.01) and TS (r = 0.46, p = 0.04).

**Discussion:**

In healthy participants, PPT and CPM did not significantly differ after a leg extension exercise performed at a high intensity, low intensity, or quiet rest condition. It is possible pre-intervention CPM testing with a noxious stimuli may have impaired inhibitory effects frequently observed during exercise but future research would need to examine this hypothesis.

## Introduction

An acute bout of exercise may produce a lessening of pain sensitivity, termed Exercise-Induced Hypoalgesia (EIH). In healthy individuals, resistance exercise consistently produces small to moderate EIH *[1, 2]*. A large body of literature has examined EIH effects during isometric exercise and, as a result, a systematic review with meta-analysis identified the optimal intensity to produce hypoalgesia: low-to-moderate intensity contractions held for a longer duration ((50% maximum voluntary contraction (MVC) until task failure, typically ~5–9 minutes)) *[1]*. However, due to a limited number of studies examining EIH effects during dynamic resistance exercise, dosing parameters to produce the largest hypoalgesia during dynamic resistance exercise remain unknown *[1, 2]*.

An individual’s response to exercise is modulated by a complex interaction of biopsychosocial factors and exercise parameters, such as intensity. As a result, it becomes reasonable to conduct a randomized controlled trial to investigate how varying dynamic resistance exercise intensities impact EIH and explore if baseline pain sensitivity factors are associated this response. The results are clinically relevant as dynamic resistance exercise is frequently prescribed in rehabilitation of patients with musculoskeletal pain. Randomized controlled trials have been previously implemented to examine how pain perception is altered after aerobic exercise (three treadmill running intensities) *[3]*. In this study, participants randomly assigned to the moderate-to-low intensity running group produced significantly higher pressure pain thresholds than the high intensity group *[3]*. A randomized controlled trial design has the benefit of directly comparing exercise intensities against a control group while also controlling for systematic differences that may affect the outcome. However, this methdology has not been previously applied to dynamic resistance exercise.

Previous dynamic resistance exercise research has implemented a crossover design and, as a result, few randomized controlled trials have been conducted in this type of exercise. To examine the effects of dynamic resistance exercise on the perception of pain, Koltyn and Arbogast conducted a crossover trial in which thirteen healthy participants completed a resistance exercise at 75% of an individual’s one repetition maximum (1RM) and a quiet rest condition in separate sessions *[4].* Focht and Koltyn conducted a crossover trial of twenty-one healthy adults that completed dynamic resistance exercise at 75% 1RM to examine time of day effects *[5].* While these studies demonstrated strong methodology, they did not directly compare resistance exercise intensity. Current evidence on optimal intensity for dynamic resistance exercise is limited as prior studies with pain threshold as the outcome have only included dynamic resistance exercise performed at 75% of the 1RM *[1, 2, 4, 5]*. As a result, it remains unknown how EIH is impacted by low or high intensity during a dynamic resistance exercise.

While pain thresholds may be the most frequently implemented outcome to measure EIH in a healthy population, changes in dynamic QST measures, such as CPM, may also be observed. After an acute bout of isometric knee extension (30% MVC until task failure), CPM improved in healthy participants (mean ± standard deviation percent change = 13.7± 19.1%) *[6]*. CPM response improves after low-to-moderate intensity running but is impaired after high intensity treadmill running, indicating intensity of exercise may impact endogenous pain modulation *[3]*. However, examining if CPM changes during different dynamic resistance exercise intensities has not been examined.

In addition to examining the effects of exercise intensity, baseline pain sensitivity factors that may modulate response to exercise should be considered in EIH trials. It remains unclear why certain individuals demonstrate hyperalgesia in response to exercise; *[1, 2]* however, pain sensitization may be a contributing factor. Significantly higher temporal summation and lower pressure pain threshold at multiple locations have been demonstrated in individuals who are considered ‘non-responders’ to a twelve week aerobic and strength training in patients with knee osteoarthritis *[7]*. Temporal summation is also predictive of individuals who display an increase in clinical pain during exercise *[8]*. Examining the association between baseline pain sensitivity and response to different dynamic resistance exercise intensities could provide preliminary evidence of factors contributing to hypoalgesia during dynamic resistance exercise.

Therefore, the primary purpose of this randomized controlled trial is to compare PPT applied over the quadriceps and trapezius during three interventions: high intensity dynamic resistance exercise, low intensity exercise, and quiet rest. We hypothesized that, based on the results in isometric exercise, low to moderate intensity exercise would produce the greatest hypoalgesia. The second purpose of this study is to examine if CPM changes after these interventions. We hypothesized, based on data from aerobic exercise, that CPM would significantly improve after low intensity resistance training compared to other groups. The third purpose is to examine if baseline pain sensitivity measures are associated with the magnitude of EIH during the interventions. We hypothesized temporal summation would be associated with EIH. This research helps to fill an important gap in rehabilitation literature as resistance exercise is a recommended intervention for patients with musculoskeletal pain. However, dosing guidelines are not available due to a lack of randomized controlled trials *[9–12]*.

## Methods

### Participants

Sixty healthy participants between 18–60 years old enrolled in the study. Participants were excluded for: regular use of prescription pain medications, current or history of chronic pain, use of blood-thinning medication, systemic medical conditions known to affect sensation, contraindications to the application of ice, presence of cardiovascular, pulmonary, or metabolic disease, use of tobacco products, not physically ready to exercise without a medical exam indicated by the Physical Activity Readiness Questionnaire Plus (PAR-Q+), or surgery, injury, or fracture to the lower back or lower extremity within the past six months. This study was approved by the University of Central Florida Institutional Review Board for Human Subjects Research and prospectively registered with clinicaltrials.gov (NCT05561582). All participants provided written informed consent to enroll.

### Experimental procedures

**Fig 1:**
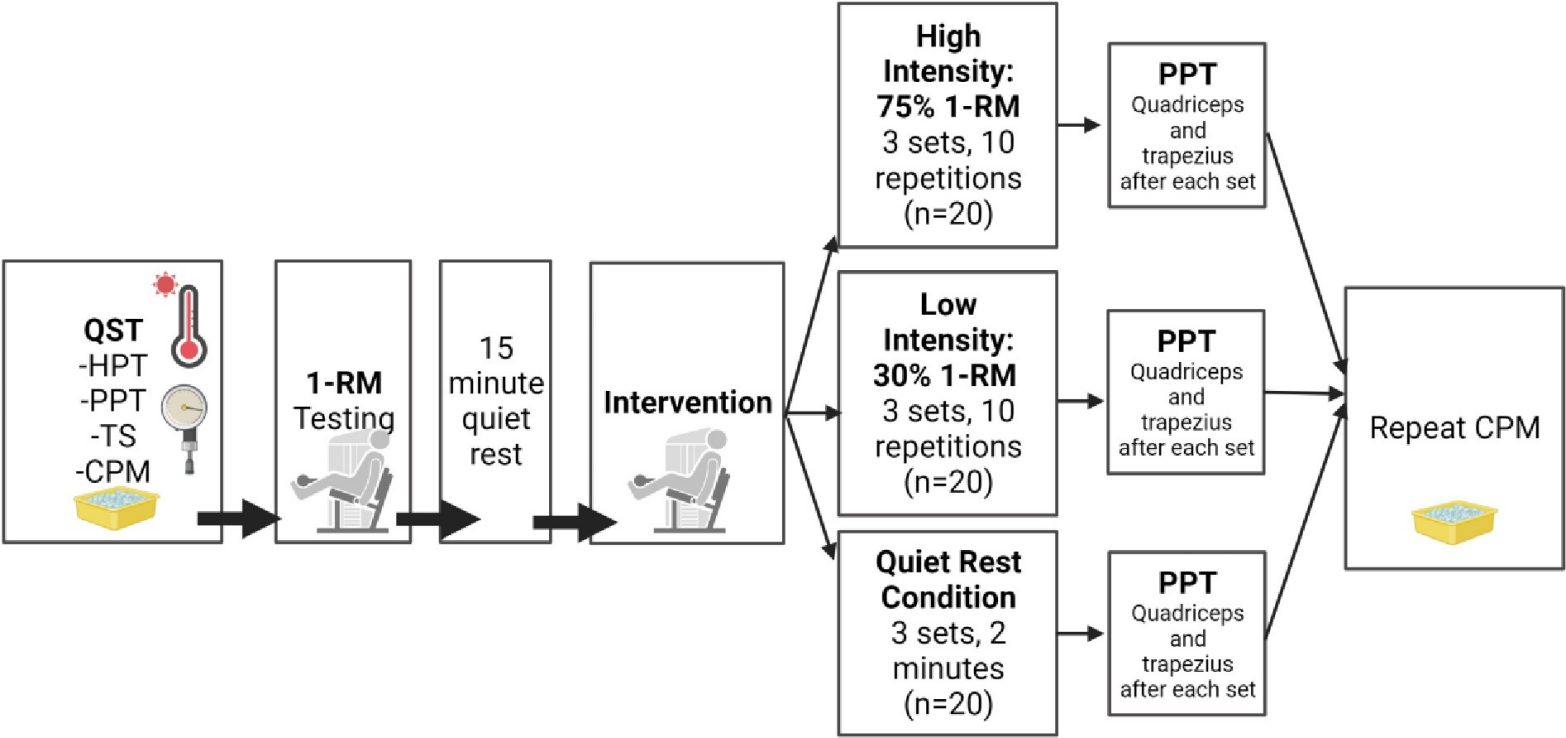
Overview of randomized controlled trial. Note: QST–Quantitative Sensory Testing, HPT–Heat Pain Threshold, PPT–Pressure Pain Threshold, CPM–Conditioned Pain Modulation. PPT was performed on the trapezius and quadriceps on the same side of the body as the exercise (dominant side). 1-RM– 1 Repetition Maximum Testing. Knee extension exercises were performed for 3 sets x 10 repetitions on the dominant leg. Figure created with Biorender.com.

This is a randomized controlled trial in which participants attended one testing session consisting of the procedures outlined in **Fig 1.**

After consenting to participate, participants reported demographic characteristics (sex, age, race, ethnicity) with a standard intake form. Participants also self-reported the minutes per week of exercise on this form. Next, participants were trained on rating pain using a standardized script and underwent a familiarization protocol with the experimental thermal and heat stimuli. Pain was rated during QST using a 101-point Numeric Pain Rating Scale (NPRS) in which 0 = no pain and 100 = most intense pain sensation imaginable *[13–16]*. Next, participants underwent a battery of Quantitative Sensory Testing for the purpose of a multi-modal pain assessment. First, participants completed thermal pain testing consisting of Heat Pain Threshold (HPT) and Temporal Summation (TS). Thermal pain testing was conducted for two purposes. First, thermal pain testing was used to determine if the intervention groups differed by pain sensitivity factors. Second, to accomplish the third purpose of the study, baseline HPT and TS were correlated with EIH magnitude to determine if these factors are associated with response to exercise.

During HPT testing, a 2 x 1-inch thermode attached to a TCS-II (QST.Lab, Strasbourg, France) increased by 1 degree Celsius (°C)/second from a baseline of 32°C to a maximum of 50°C. Participants pressed a button when the sensation changed from “comfortable warmth to slightly unpleasant pain.” Two trials were completed with the average temperature analyzed as this improves reliability *[17]*. For TS, ten heat pulses peaking at 49°C with a standard inter-stimulus interval were applied to the palmar surface of the non-dominant hand *[18]*. During each heat pulse, participants rated the magnitude of their “second pain” with the NPRS. TS was calculated as the: NPRS on the fifth pulse–NPRS on the first pulse *[19]*.

After thermal pain testing, participants underwent baseline pressure pain threshold (PPT) testing. PPT was conducted prior to the intervention to determine if the intervention groups differed by pain sensitivity factors and to test if the washout period after the 1 repetition maximum test was sufficient. During PPT, a computerized pressure algometer (AlgoMed, Ramat Yishai, Israel) with a 1 cm diameter rubber tip was applied at a constant rate. Participants pressed a button when the sensation changed from “comfortable pressure to slightly unpleasant pain.” Two trials were completed with the average threshold in kilopascals (kPa) analyzed. Inter-rater reliability is excellent in healthy participants and measurement error is reduced with two trials *[20]*.

Finally, participants completed pre-intervention Conditioned Pain Modulation (CPM) testing to allow for examination of our secondary aim. CPM is a psychophysical measure of an endogenous pain inhibitory process in which two noxious stimuli are applied (e.g. pressure to the foot and cold water to the hand) *[21]*. This ‘pain inhibits pain’ phenomenon is a descending modulatory process potentially mediated by the spino-bulbo-spinal loop *[22]*. The testing stimulus (PPT) was applied to the web space of the non-dominant foot for two trials with the average analyzed as the first testing stimulus. Participants then immersed the dominant hand into water cooled by a refrigeration unit (ARCTIC Series Refrigerated Bath Circulator, ThermoFisher Scientific, Massachusetts, USA) that circulated water continuously to maintain a constant temperature of 12°C for 60 seconds. Immediately following the cold water immersion, the testing stimulus was repeated on the foot. Participants rested quietly for 15 minutes after CPM testing to allow pain sensitivity changes to normalize before exercise testing *[23]*. This combination of pressure as a testing stimulus and cold pressor test at 12°C was selected due to the highest inter-session reliability (ICC = 0.77, 95% CI 0.42–0.59) in healthy participants *[24]*.

### 1-Repetition maximum (1RM)

1RM testing of leg extension was completed on the dominant side in a Steelflex Plate Loaded Single Leg Extension machine (Taipei, Taiwan). Participants performed a brief warm up consisting of 5–10 repetitions with a light resistance followed by a 1-minute rest period. The researcher estimated the amount of weight the participant could lift 3–5 times before failure. Participants then completed a 5RM test followed by a two-minute rest break. The researcher then estimated the near-maximal load that would allow the participant to complete 2–3 repetitions and the participant completed a 2RM test. The participant was provided another two-minute rest break then attempted the 1RM. If the participant did not reach maximal effort, the participant was provided an additional two-minute rest break and the load was increased. This was repeated a maximum of three times [25]. This protocol adheres to the 1RM testing recommendations from the National Strength and Conditioning Association [25] and all participants reached the 1RM within three attempts. The participant’s 1RM was used to determine the amount of weight to add to the knee extension machine for the intervention. The 1RM of seated knee extension on one leg demonstrates excellent intra and inter-rater reliability (ICC = 0.96, measurement error = 3.2 kg) [26]. After 1RM testing, participants rested quietly for 15 minutes to allow for any exercise-related inhibitory effects to normalize prior to the intervention [27].

### Intervention

Participants were randomly assigned to receive one of the following three interventions: 1) high intensity leg extension exercise with weight equivalent to 75% of the 1RM (3 sets, 10 repetitions), 2) low intensity leg extension exercise with weight equivalent to 30% of the 1RM (3 sets, 10 repetitions), or 3) quiet rest (2 minutes of quiet rest, 3 times) condition. All exercise interventions were performed on the same SteelFlex machine that was used for the 1RM testing *[28]*. Participants were randomly assigned to the intervention using the research website randomizer.org prior to the first participant enrolling and intervention assignments were maintained in an opaque envelope.

To examine the first aim of our study, PPT was applied to the dominant quadriceps and upper trapezius for four total testing sets: before set 1, after set 1, after set 2, after set 3. These sites were selected as they are consistent with prior EIH studies involving isometric quadriceps exercise *[29, 30]* demonstrating exercise may produce local and remote changes in pain sensitivity *[31,32]*. To ensure consistency in the site of application, stimuli were applied halfway between the hip crease and superior border of the patella for the quadriceps *[33]* and halfway between the C7 spinous process and acromion for the trapezius *[34]*.

Immediately after the intervention, all participants repeated CPM with the previously described protocol to accomplish the second aim of our study. CPM testing was conducted after the final PPT measurement for EIH.

### Statistical analysis

#### Sample size calculation

The power analysis was conducted based on the first aim of the study. G-power v.3.1.9.7 analysis for a repeated-measures ANOVA with a within-between interaction indicated 60 participants provided 80% power with alpha level = 0.05 to determine time (4 measurements) x intervention (3 groups) interaction effects with a small effect size (partial eta^2^ = 0.03).

#### Sample characteristics

IBM SPSS version 28 (Armonk, NY) was used for statistical analyses. Descriptive statistics, including demographic factors of the total sample and by group, were calculated. A one-way ANOVA examined for differences in demographic and pain sensitivity factors between groups. Probability was set at an alpha level of 0.05 for each analysis test and all results are presented as mean ± standard deviation.

#### PPT after washout period

To examine if the PPT returned to baseline after 1RM testing, a repeated measures ANOVA examined for differences in PPT at the quadriceps and trapezius measured at baseline and at the start of the intervention.

#### Compare PPT changes by group

To compare immediate changes in PPT at a local and distant site by intervention, a three-way ANOVA examined for within-subject factors of time (PPT before, after set 1, 2, 3) x site (upper trapezius and quadriceps) x between subject factor of intervention assignment (quiet rest, low intensity, high intensity). Simple effects decomposition with Bonferroni correction was performed.

#### Changes in CPM after each intervention

Absolute CPM was calculated with the following formula: First Testing Stimulus–Second Testing Stimulus *[35]*. To examine if CPM changed after dynamic resistance, a mixed model repeated measures ANOVAs examined for time (pre and post intervention CPM) x intervention (low, high intensity, quiet rest) interaction effects. Simple effects decomposition with Bonferroni correction was performed.

#### Correlate baseline pain sensitivity with EIH during exercise

A Pearson Correlation Coefficient examined the association between baseline pain sensitivity factors (HPT, TS, CPM) with EIH. EIH was calculated with following formula: PPT after the third set–PPT before the first set (after washout period).

## Results

Sixty participants completed the study, demonstrated in the CONSORT Flow Diagram below. Data collection occurred October 31, 2022 –March 27, 2023.

Insert CONSORT Diagram (this is part of the uploaded files)

As demonstrated in **Table 1**, the intervention groups did not significantly differ by demographic factors (p’s>0.05), 1RM, or minutes per week of exercise. As demonstrated in **Table 2**, baseline pain sensitivity measures also did not differ by intervention group (p’s>0.05).

**Table 1:** Demographic characteristics of the total sample and by intervention assignment.

	Total Sample (n = 60)	Low Intensity Exercise (n = 20)	High Intensity Exercise (n = 20)	Quiet Rest (n = 20)	p-value
**Age**	21.36 ± 2.27	21.50 ± 2.19	20.89 ± 1.74	21.60 ± 2.76	0.57
**Sex (% female)**	81.70	75.00	75.00	95.0	0.26
**Race**					0.86
**% Asian**	6.70	5.00	5.00	10.00	
**% African**					
**American**	15.00	10.00	20.00	15.00	
**% White**	78.30	85.00	75.00	75.00	
**Ethnicity (% Hispanic)**	26.70	25.00	35.00	20.00	0.59
**Minutes per week of exercise**	242.39 ± 187.49	285.08 ± 166.60	250.85 ± 226.55	191.25 ± 159.18	0.28
**1-repetition maximum (pounds)**	65.10 ± 23.37	66.68 ± 17.79	64.35 ± 27.78	64.31 ± 24.38	0.94

Note: Values reported represent mean ± standard deviation. Alpha level was set at p <0.05 for statistical significance.

* Indicates statistical significance.

**Table 2:** Baseline pain sensitivity characteristics of the total sample and by intervention assignment.

	Total Sample (n = 60)	Low Intensity Exercise (n = 20)	High Intensity Exercise (n = 20)	Quiet Rest (n = 20)	p-value
**HPT–Quadriceps temperature (** ^**o**^ **C)**	42.99 ± 1.89	43.26 ± 1.85	42.71 ± 2.00	43.01 ± 1.89	0.67
**HPT–Trapezius temperature (** ^**o**^ **C)**	43.11 ± 1.98	42.79 ± 1.74	43.15 ± 2.30	43.38 ± 1.91	0.65
**PPT-Quadriceps pressure (kPa)**	485.19 ± 165.92	496.19 ± 175.19	499.83 ± 183.34	459.58 ± 141.85	0.71
**PPT-Trapezius pressure (kPa)**	332.89 ± 121.17	341.56 ± 133.53	342.93 ± 131.36	314.19 ± 99.77	0.71
**TS**	6.74 ± 11.98	2.85 ± 6.63	8.20 ± 10.37	9.31 ± 16.63	0.19
**CPM**	-47.63 ± 72.19	-38.85 ± 65.85	-49.99 ± 73.38	-54.03 ± 79.56	0.79
**CPM (% Efficient)**	70.00	70.00	65.00	75.00	0.78

Note: Values reported represent mean ± standard deviation. Alpha level was set at p <0.05 for statistical significance.

* Indicates statistical significance. °C = Degrees Celsius, NPRS = numeric pain rating scale, kPa = kilopascals, HPT = Heat Pain Threshold, PPT = Pressure Pain Threshold, TS = Temporal Summation, CPM = Conditioned Pain Modulation

### Compare PPT changes by group

*PPT after washout period*. PPT applied over the quadriceps and trapezius did not significantly differ from baseline to the start of the intervention suggesting the washout period after 1RM testing was sufficient (quadriceps p = 0.49, trapezius p = 0.11).

*Compare PPT changes by group*. Mauchly’s test was significant for PPT (chi-square(5) = 18.32, p<0.01) and time x site interaction (chi-square(5) = 19.67, p<0.01). Estimates of sphericity were greater than 0.75 and, therefore, Huynh-Feldt correction was used (Epsilon: time = 0.87, time x site = 0.88) [36]. Levene’s test was non-significant (p’s>0.05), indicating homogeneity of variance assumption was met.

Time x site x intervention interaction effects were not significant (F(5.3, 150.97) = 0.87, p = 0.51, partial eta^2^ = 0.03). Time x site interaction effects were not significant (F(2.65, 57), p = 0.06, partial eta^2^ = 0.04). Site x intervention interaction effects were not significant (F(2, 57), p = 0.07, partial eta^2^ = 0.09). This suggests PPT did not significantly differ between groups.

**Fig 2:**
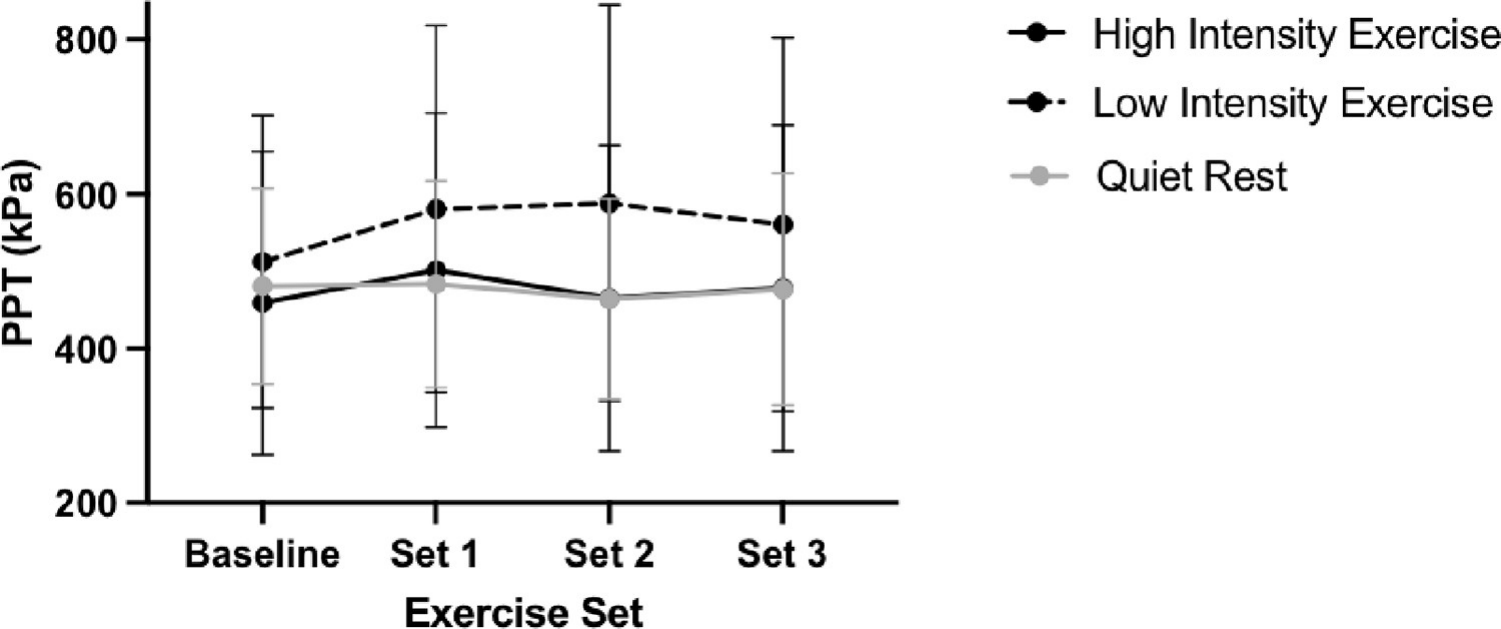
Immediate effects of PPT changes in the quadriceps. Note: Error bars represent standard deviation.

**Fig 3:**
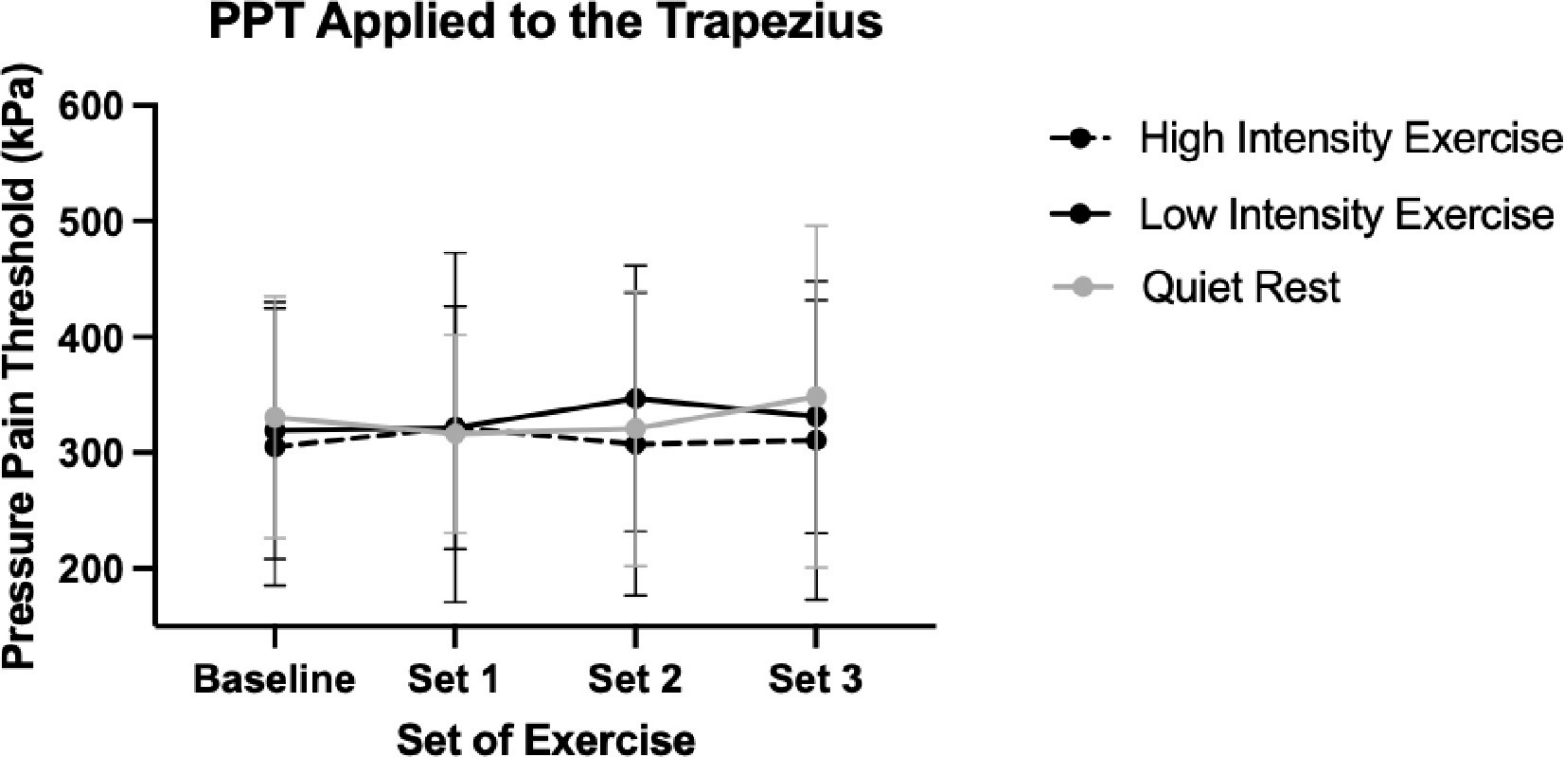
Immediate effects of PPT changes in the trapezius. Note: Error bars represent standard deviation.

Time x intervention interaction effects trended toward significance (F(5.23, 149.15) = 2.25, p = 0.05, partial eta^2^ = 0.07). A main effect of site was significant (F(1,57) = 141.76, p<0.01, partial eta^2^ = 0.71) with the quadriceps displaying significantly higher PPT compared to the trapezius (mean difference = 180.99, SE = 15.20 kPa). A main effect of time was not significant F(2.62, 149.15) = 2.12, p = 0.11, partial eta^2^ = 0.04). Changes in PPT by intervention for each site can be found in **Figs 2 and 3.**

### Changes in CPM after each intervention

CPM did not significantly change after the interventions. Time x Intervention interaction effects were not significant (F(1,38) = 0.81, p = 0.37, partial eta^2^ = 0.02. Main effects of time were also not observed F(2,57) = 0.76, p = 0.47, partial eta2 = 0.03).

### Correlate baseline pain sensitivity with EIH during exercise low intensity

As demonstrated in **Table 3,** EIH effects at the quadriceps displayed a significant, positive moderate association with baseline HPT applied over the trapezius (r = 0.61, p<0.01) and TS (r = 0.46, p = 0.04). Additional baseline pain sensitivity measures were not significantly correlated with EIH response during low intensity exercise.

**Table 3:** Correlation between baseline pain sensitivity and pressure pain threshold changes during each intervention.

	Heat Pain Threshold Temperature (°C) Quadriceps		Heat Pain Threshold Temperature (°C) Trapezius		Temporal Summation		Conditioned Pain Modulation	
	r	p-value	r	p-value	r	p-value	r	p-value
**Low Intensity**								
EIH Quadriceps	0.35	0.14	0.61	<0.01*	0.46	0.04*	-0.08	0.75
EIH Trapezius	0.42	0.07	0.39	0.09	0.08	0.73	-0.19	0.41
**High Intensity**								
EIH Quadriceps	-0.12	0.63	-0.05	0.85	-0.19	0.43	-0.28	0.24
EIH Trapezius	-0.33	0.17	-0.09	0.68	0.15	0.52	0.23	0.33
**Quiet Rest**								
PPT Quadriceps	-0.04	0.89	0.25	0.29	-0.07	0.78	-0.06	0.81
PPT Trapezius	-0.16	0.51	-0.12	0.62	-0.12	0.63	-0.37	0.11

Note: EIH = Exercise Induced Hypoalgesia, Calculated as the difference between the pre and post exercise pressure pain threshold. PPT = Pressure Pain Threshold, Calculated as the difference between pre and post PPT after quiet rest. r = Pearson Correlation Coefficient. p<0.05 is statistically significant as indicated by *.

### High intensity

As demonstrated in **Table 3**, baseline pain sensitivity was not significantly associated with EIH response at either site during high intensity exercise.

### Quiet rest

As demonstrated in **Table 3**, baseline pain sensitivity was not significantly associated with changes in PPT at either site during the control condition.

## Discussion

Limited research has examined the magnitude of EIH effects by dose during a dynamic resistance exercise [1, 2]. This randomized controlled trial suggests that, in healthy participants, PPT did not significantly differ between groups (high intensity, low intensity, or control condition). Furthermore, CPM did not significantly change after each intervention. In examining baseline pain sensitivity factors that may be associated with response to each intervention, significant correlations were not observed with the exception of a significant, positive moderate association with baseline HPT applied over the trapezius and TS with EIH at the quadriceps during low intensity exercise.

The first finding of the study is that PPT did not significantly differ between groups (high intensity, low intensity, or control condition). 30% 1RM was selected as this is considered a low intensity resistance training dose [37]. Prior research has also demonstrated knee extension dynamic resistance exercise for three sets of ten repetitions at 75% 1RM produced significant hypoalgesia at the hand [4, 5] compared to quiet rest [5]. While we anticipated the intensity of exercise would produce differences in the perception of pain, our results suggest that intensity based on a 1 repetition maximum does not impact immediate changes in PPT.

Two important methodological differences between our work and previously published studies in dynamic resistance exercise [4, 5] should be noted. First, participants in our study completed a single leg knee extension protocol consistent with exercise prescription for an individual recovering from a unilateral lower extremity injury. We selected the dominant leg for this study in healthy participants to standardize exercise location across individuals. Second, we applied PPT at the quadriceps and upper trapezius whereas prior studies [4, 5] applied it to the middle digit of the left forefinger. Given the evidence supporting local and distant hypoalgesia effects of isometric exercise, our team decided to modify testing sites. Some of these methodological differences [4, 5, 38] may contribute our results.

An additional methodological difference is that, although we provided a washout period, we hypothesize applying CPM testing prior to the exercise intervention may have impaired pain inhibitory capacity during exercise. This similar effect was reported in a prior study [29] in which baseline CPM testing diminished systemic EIH effects [29]. The application of a noxious stimuli, such as CPM, prior to exercise may remove the inhibitory effects of this intervention [39]. Individuals who underwent a cold pressor task prior to fifteen minutes of bicycling at a high intensity (75% VO2 max) did not display an increase in PPT after exercise, suggesting EIH was attenuated after CPM activation [39]. CPM activation prior to exercise may have attenuated EIH effects. It is possible exercise related discomfort may be a noxious stimuli capable of eliciting a CPM effect yet this would need to be tested in future trials. Shared underlying mechanisms between CPM and EIH [29, 30, 40] have been previously reported as baseline CPM predicts EIH in healthy adults [40].

While our study found that an acute bout of high intensity or low intensity exercise did not significantly change PPT, it is also possible that multiple sessions are needed to see an effect. A prior randomized controlled trial in patients with chronic low back pain compared a high-intensity to a moderate-intensity aerobic training on a cycle ergometer and resistance training circuit (three upper body, three lower body exercises) program for two times/week, 1.5 hours each (twenty-four total sessions).[41] Similar to the leg extension protocol used in our study, the resistance training circuit consisted of dynamic resistance exercises completed with exercise machines in a gym. Individuals in the high intensity group demonstrated greater improvements in disability and exercise capacity than those in the moderate intensity group.[41] Future trials may aim to conduct a longitudinal study to determine the long term effects of a repeated high intensity versus low-moderate intensity protocol.

The second result of this study is CPM did not significantly change after dynamic resistance exercise or quiet rest. Repeated CPM testing may result in reduced efficiency on the second trial [19]; however, we were interested in the effects of the intervention on CPM. In looking at the EIH literature, changes in CPM after exercise may depend if an individual is an EIH responder. Only systemic EIH responders to isometric knee extension exercise demonstrate a reduction in CPM after exercise [29]. Furthermore, an additional explanation for why CPM did not change may be related to the CPM methods. In our trial, a sequential testing method for CPM paradigm was implemented. However, a parallel method results in a slightly higher CPM effect in healthy individuals with a mechanical test stimuli.[42] While CPM did not significantly change after dynamic resistance exercise, future trials may aim to test CPM using a parallel method.

The third result of this study was investigating personal characteristics associated with the response to dynamic resistance exercise as a first step in identifying indivduals likely to have favorable reductions in pain. In examining baseline pain sensitivity factors that may be associated with response to each intervention, significant correlations were not observed with the exception of a significant, positive moderate association with baseline heat pain threshold applied over the trapezius (r = 0.61, p<0.01) and temporal summation (r = 0.46, p = 0.04) with EIH during low intensity exercise. Temporal summation has been previously associated with response to exercise[7, 8]. However, our study demonstrated this measure is also associated with response to low intensity dynamic resistance exercise in healthy individuals which contributes to the literature in a novel way and expands our broader understanding. This suggests that individuals with elevated central mechanisms may have greater response to low intensity exercise. Prior research supports that individuals with nociplastic pain conditions characterized by elevated temporal summation[18] demonstrate the greatest benefit from low to moderate intensity exercise.[43] An additional novel finding of this study is that a higher heat pain threshold at a distant site is moderately correlated with greater EIH during low intensity exercise. Heat pain threshold and temporal summation are both C-fiber mediated, suggesting this pathway may be associated with response to low intensity exercise. [44, 45]

### Limitations

The limitations of this study should be considered when interpreting the results. While prior EIH studies have reported inhibitory effects for up to thirty minutes after maximal aerobic exercise [46], we selected a shorter washout period of fifteen minutes. Furthermore, we did not specifically recruit for or exclude participants based on training status which can impact pain sensitivity and EIH response. We also had a small sample of individuals who did not have a CPM response.

## Conclusions

As part of a randomized controlled trial, healthy participants underwent baseline pain sensitivity testing followed by random assignment to one of three interventions: high intensity dynamic resistance exercise (75% 1RM), low intensity exercise (30% 1RM), or quiet rest control. Primary outcomes included PPT applied over the trapezius and quadriceps during each set of exercise and pre/post CPM. The groups did not significantly differ by PPT or CPM.

## Supporting information

S1 Fig (TIF)

S1 Data (XLSX)

## References

[1] Naugle KM, Fillingim RB, Riley J. L. 3rd. A meta-analytic review of the hypoalgesic effects of exercise. J Pain. 2012;13: 1139–1150. doi: 10.1016/j.jpain.2012.09.006 23141188 PMC3578581

[2] Wewege MA, Jones MD. Exercise-Induced Hypoalgesia in Healthy Individuals and People With Chronic Musculoskeletal Pain: A Systematic Review and Meta-Analysis. J Pain. 20200626th ed. 2021;22: 21–31. doi: 10.1016/j.jpain.2020.04.003 32599154

[3] Zi-Han X, Nan A, Rui CJ, Yong-Long Y. Modulation of pain perceptions following treadmill running with different intensities in females. Physiol Rep. 2023;11: e15831. doi: 10.14814/phy2.15831 37749050 PMC10519819

[4] Koltyn KF, Arbogast RW. Perception of pain after resistance exercise. Br J Sports Med. 1998;32: 20–4. doi: 10.1136/bjsm.32.1.20 9562159 PMC1756063

[5] Focht BC, Koltyn KF. Alterations in pain perception after resistance exercise performed in the morning and evening. J Strength Cond Res. 2009;23: 891–7. doi: 10.1519/JSC.0b013e3181a05564 19387388

[6] Alsouhibani A, Hoeger Bement M. Impaired conditioned pain modulation was restored after a single exercise session in individuals with and without fibromyalgia. Pain Rep. 20220401st ed. 2022;7: e996. doi: 10.1097/PR9.0000000000000996 35399187 PMC8984585

[7] Hattori T, Ohga S, Shimo K, Niwa Y, Tokiwa Y, Matsubara T. Predictive Value of Pain Sensitization Associated with Response to Exercise Therapy in Patients with Knee Osteoarthritis: A Prospective Cohort Study. J Pain Res. 2022;15: 3537–3546. doi: 10.2147/JPR.S385910 36394057 PMC9653041

[8] Vaegter HB, Petersen KK, Sjodsholm LV, Schou P, Andersen MB, Graven-Nielsen T. Impaired exercise-induced hypoalgesia in individuals reporting an increase in low back pain during acute exercise. Eur J Pain. 20210117th ed. 2021;25: 1053–1063. doi: 10.1002/ejp.1726 33400333

[9] George SZ, Fritz JM, Silfies SP, Schneider MJ, Beneciuk JM, Lentz TA, et al. Interventions for the Management of Acute and Chronic Low Back Pain: Revision 2021. Journal of Orthopaedic & Sports Physical Therapy. 2021;51: CPG1–CPG60. doi: 10.2519/jospt.2021.0304 34719942 PMC10508241

[10] Oliveira CB, Maher CG, Pinto RZ, Traeger AC, Lin CC, Chenot JF, et al. Clinical practice guidelines for the management of non-specific low back pain in primary care: an updated overview. Eur Spine J. 20180703rd ed. 2018;27: 2791–2803. doi: 10.1007/s00586-018-5673-2 29971708

[11] Willy RW, Hoglund LT, Barton CJ, Bolgla LA, Scalzitti DA, Logerstedt DS, et al. Patellofemoral Pain. J Orthop Sports Phys Ther. 2019;49: Cpg1–cpg95. doi: 10.2519/jospt.2019.0302 31475628

[12] Martin RL, Chimenti R, Cuddeford T, Houck J, Matheson JW, McDonough CM, et al. Achilles Pain, Stiffness, and Muscle Power Deficits: Midportion Achilles Tendinopathy Revision 2018. J Orthop Sports Phys Ther. 2018;48: A1–a38. doi: 10.2519/jospt.2018.0302 29712543

[13] Bolton JE, Wilkinson RC. Responsiveness of pain scales: a comparison of three pain intensity measures in chiropractic patients. J Manipulative Physiol Ther. 1998;21: 1–7. 9467094

[14] Hartrick CT, Kovan JP, Shapiro S. The numeric rating scale for clinical pain measurement: a ratio measure? Pain Pract. 2003;3: 310–316. doi: 10.1111/j.1530-7085.2003.03034.x 17166126

[15] Jensen MP, Turner JA, Romano JM, Fisher LD. Comparative reliability and validity of chronic pain intensity measures. Pain. 1999;83: 157–162. doi: 10.1016/s0304-3959(99)00101-3 10534586

[16] Jensen MP, Karoly P, Braver S. The measurement of clinical pain intensity: a comparison of six methods. Pain. 1986;27: 117–126. doi: 10.1016/0304-3959(86)90228-9 3785962

[17] Moloney NA, Hall TM, Doody CM. Reliability of thermal quantitative sensory testing: a systematic review. J Rehabil Res Dev. 2012;49: 191–207. doi: 10.1682/jrrd.2011.03.0044 22773522

[18] Price DD, Staud R, Robinson ME, Mauderli AP, Cannon R, Vierck CJ. Enhanced temporal summation of second pain and its central modulation in fibromyalgia patients. Pain. 2002;99: 49–59. doi: 10.1016/s0304-3959(02)00053-2 12237183

[19] Valencia C, Fillingim RB, George SZ. Suprathreshold heat pain response is associated with clinical pain intensity for patients with shoulder pain. Journal of Pain. 2011;12: 133–140. doi: 10.1016/j.jpain.2010.06.002 20692209 PMC3697912

[20] Mailloux C, Beaulieu LD, Wideman TH, Massé-Alarie H. Within-session test-retest reliability of pressure pain threshold and mechanical temporal summation in healthy subjects. PLoS One. 20210112th ed. 2021;16: e0245278. doi: 10.1371/journal.pone.0245278 33434233 PMC7802960

[21] Pud D, Granovsky Y, Yarnitsky D. The methodology of experimentally induced diffuse noxious inhibitory control (DNIC)-like effect in humans. Pain. 2009;144: 16–19. doi: 10.1016/j.pain.2009.02.015 19359095

[22] Millan MJ. Descending control of pain. Progress in neurobiology. 2002;66: 355–474. doi: 10.1016/s0301-0082(02)00009-6 12034378

[23] Kennedy DL, Kemp HI, Ridout D, Yarnitsky D, Rice AS. Reliability of conditioned pain modulation: a systematic review. Pain. 2016;157: 2410–2419. doi: 10.1097/j.pain.0000000000000689 27559835 PMC5228613

[24] Nuwailati R, Curatolo M, LeResche L, Ramsay DS, Spiekerman C, Drangsholt M. Reliability of the conditioned pain modulation paradigm across three anatomical sites. Scandinavian journal of pain. 2019. doi: 10.1515/sjpain-2019-0080 31812949

[25] Haff GG, Triplett NT. *Essentials of strength training and conditioning*. National Strength and Conditioning Association, 2021.

[26] Tagesson SKB, Kvist J. Intra- and interrater reliability of the establishment of one repetition maximum on squat and seated knee extension. J Strength Cond Res. 2007;21: 801–807. doi: 10.1519/R-20846.1 17685713

[27] Kemppainen P, Paalasmaa P, Pertovaara A, Alila A, Johansson G. Dexamethasone attenuates exercise-induced dental analgesia in man. Brain Res. 1990;519: 329–32. doi: 10.1016/0006-8993(90)90096-t 2168784

[28] Shimano T, Kraemer WJ, Spiering BA, Volek JS, Hatfield DL, Silvestre R, et al. Relationship between the number of repetitions and selected percentages of one repetition maximum in free weight exercises in trained and untrained men. J Strength Cond Res. 2006;20: 819–823. doi: 10.1519/R-18195.1 17194239

[29] Alsouhibani A, Vaegter HB, Hoeger Bement M. Systemic Exercise-Induced Hypoalgesia Following Isometric Exercise Reduces Conditioned Pain Modulation. Pain Med. 2019;20: 180–190. doi: 10.1093/pm/pny057 29618132 PMC7868957

[30] Vaegter HB, Handberg G, Graven-Nielsen T. Similarities between exercise-induced hypoalgesia and conditioned pain modulation in humans. Pain. 20130926th ed. 2014;155: 158–167. doi: 10.1016/j.pain.2013.09.023 24076045

[31] Jones MD, Taylor JL, Booth J, Barry BK. Exploring the Mechanisms of Exercise-Induced Hypoalgesia Using Somatosensory and Laser Evoked Potentials. Front Physiol. 2016;7: 581. doi: 10.3389/fphys.2016.00581 27965587 PMC5126702

[32] Vaegter HB, Graven-Nielsen T. Pain modulatory phenotypes differentiate subgroups with different clinical and experimental pain sensitivity. Pain. 2016;157: 1480–1488. doi: 10.1097/j.pain.0000000000000543 26963852

[33] Kosek E, Ekholm J. Modulation of pressure pain thresholds during and following isometric contraction. Pain. 1995;61: 481–486. doi: 10.1016/0304-3959(94)00217-3 7478692

[34] Walton DM, Levesque L, Payne M, Schick J. Clinical pressure pain threshold testing in neck pain: comparing protocols, responsiveness, and association with psychological variables. Phys Ther. 2014;94: 827–837. doi: 10.2522/ptj.20130369 24557645 PMC4040424

[35] Yarnitsky D, Bouhassira D, Drewes AM, Fillingim RB, Granot M, Hansson P, et al. Recommendations on practice of conditioned pain modulation (CPM) testing. Eur J Pain. 2015;19: 805–806. doi: 10.1002/ejp.605 25330039

[36] Field AP. Discovering Statistics Using IBM SPSS Statistics. 4th editio. SAGE Publications, Ltd; 2013.

[37] Carvalho L, Junior RM, Barreira J, Schoenfeld BJ, Orazem J, Barroso R. Muscle hypertrophy and strength gains after resistance training with different volume-matched loads: a systematic review and meta-analysis. Appl Physiol Nutr Metab. 2022;47: 357–368. doi: 10.1139/apnm-2021-0515 35015560

[38] The Effects of Aerobic Exercise and Strengthening Exercise on Pain Pressure Thresholds. [cited 7 Apr 2023]. Available: https://www.jstage.jst.go.jp/article/jpts/26/7/26_jpts-2013-501/_article 10.1589/jpts.26.1107 PMC4135207 25140106

[39] Gajsar H, Nahrwold K, Titze C, Hasenbring MI, Vaegter HB. Exercise does not produce hypoalgesia when performed immediately after a painful stimulus. Scand J Pain. 2018;18: 311–320. doi: 10.1515/sjpain-2018-0024 29794298

[40] Lemley KJ, Hunter SK, Bement MKH. Conditioned Pain Modulation Predicts Exercise-Induced Hypoalgesia in Healthy Adults. Medicine & Science in Sports & Exercise. 2015;47: 176–184. doi: 10.1249/MSS.0000000000000381 24870571

[41] Verbrugghe J, Agten A, Stevens S, Hansen D, Demoulin C, B OE, et al. Exercise Intensity Matters in Chronic Nonspecific Low Back Pain Rehabilitation. Med Sci Sports Exerc. 2019;51: 2434–2442. doi: 10.1249/MSS.0000000000002078 31269004

[42] Reezigt RR, Kielstra SC, Coppieters MW, Scholten-Peeters GGM. No relevant differences in conditioned pain modulation effects between parallel and sequential test design. A cross-sectional observational study. PeerJ. 2021;9: e12330. doi: 10.7717/peerj.12330 35003911 PMC8679953

[43] Klaps S, Haesevoets S, Verbunt J, Köke A, Janssens L, Timmermans A, et al. The Influence of Exercise Intensity on Psychosocial Outcomes in Musculoskeletal Disorders: A Systematic Review. Sports Health. 20220304th ed. 2022; 19417381221075354. doi: 10.1177/19417381221075354 35243924 PMC9631039

[44] Van Hees J, Gybels J. C nociceptor activity in human nerve during painful and non painful skin stimulation. J Neurol Neurosurg Psychiatry. 1981;44: 600–607. doi: 10.1136/jnnp.44.7.600 7288447 PMC491064

[45] Price DD, Mao J, Frenk H, Mayer DJ. The N-methyl-D-aspartate receptor antagonist dextromethorphan selectively reduces temporal summation of second pain in man. Pain. 1994;59: 165–174. doi: 10.1016/0304-3959(94)90069-8 7892014

[46] Hoffman MD, Shepanski MA, Ruble SB, Valic Z, Buckwalter JB, Clifford PS. Intensity and duration threshold for aerobic exercise-induced analgesia to pressure pain. Arch Phys Med Rehabil. 2004;85: 1183–7. doi: 10.1016/j.apmr.2003.09.010 15241771

