# Supplementary figures and images for: Exercise induced hypoalgesia during different intensities of a dynamic resistance exercise: A randomized controlled trial
Source: PLoS One. 2024 Apr 16;19(4):e0299481. doi: 10.1371/journal.pone.0299481 (PMC11020855; doi:10.1371/journal.pone.0299481)

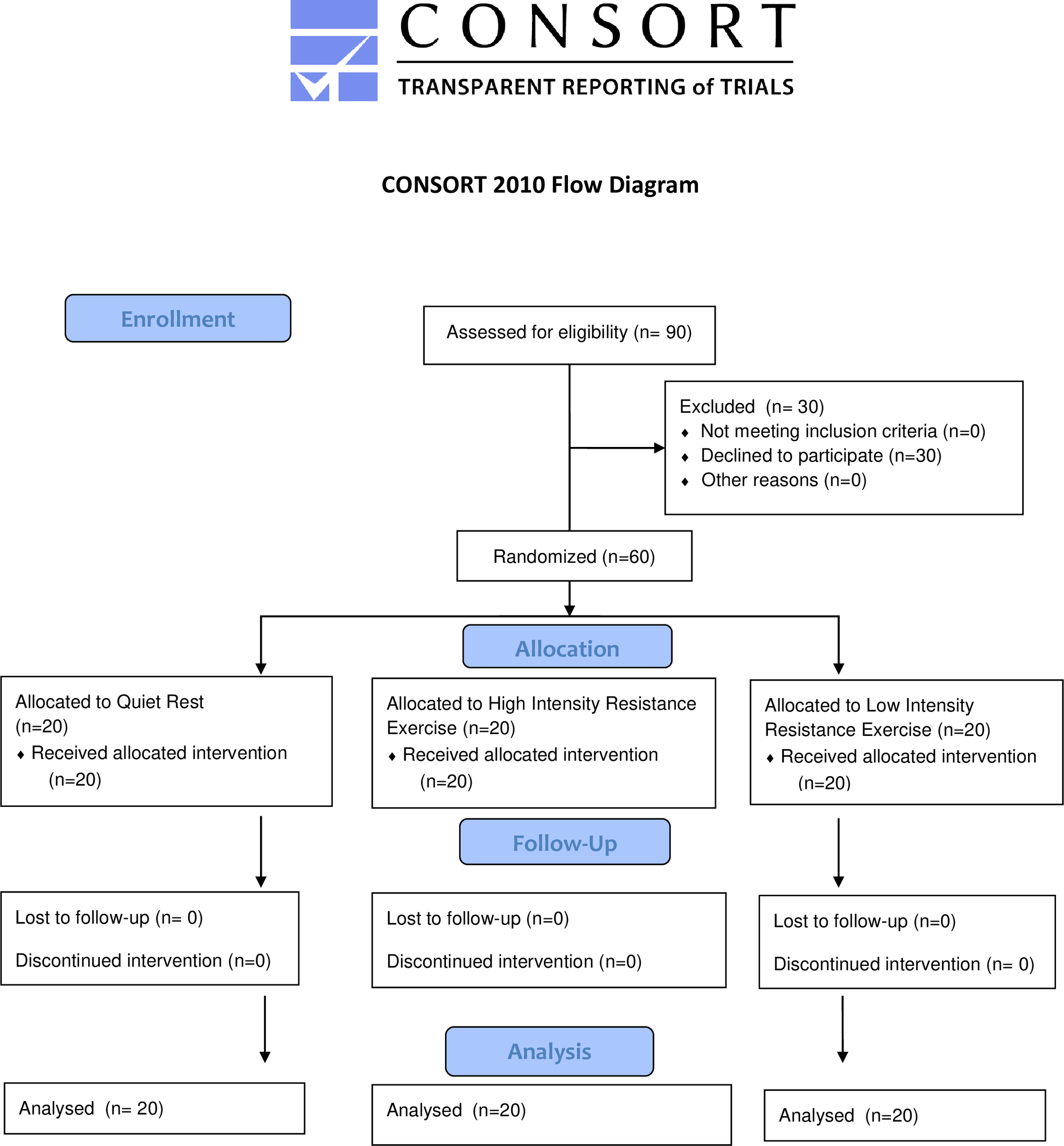

Supplement: S1 Fig — (TIF) [file pone.0299481.s001.tif]
